# Supplementary figures and images for: The Dynamic Changes in Biosynthesis and Spatiotemporal Distribution of Phytohormones Under Jasmonic Acid Treatment Provide Insights into Hormonal Regulation in Sinopodophyllum hexandrum
Source: Plants (Basel). 2025 Mar 22;14(7):1001. doi: 10.3390/plants14071001 (PMC11990078; doi:10.3390/plants14071001)

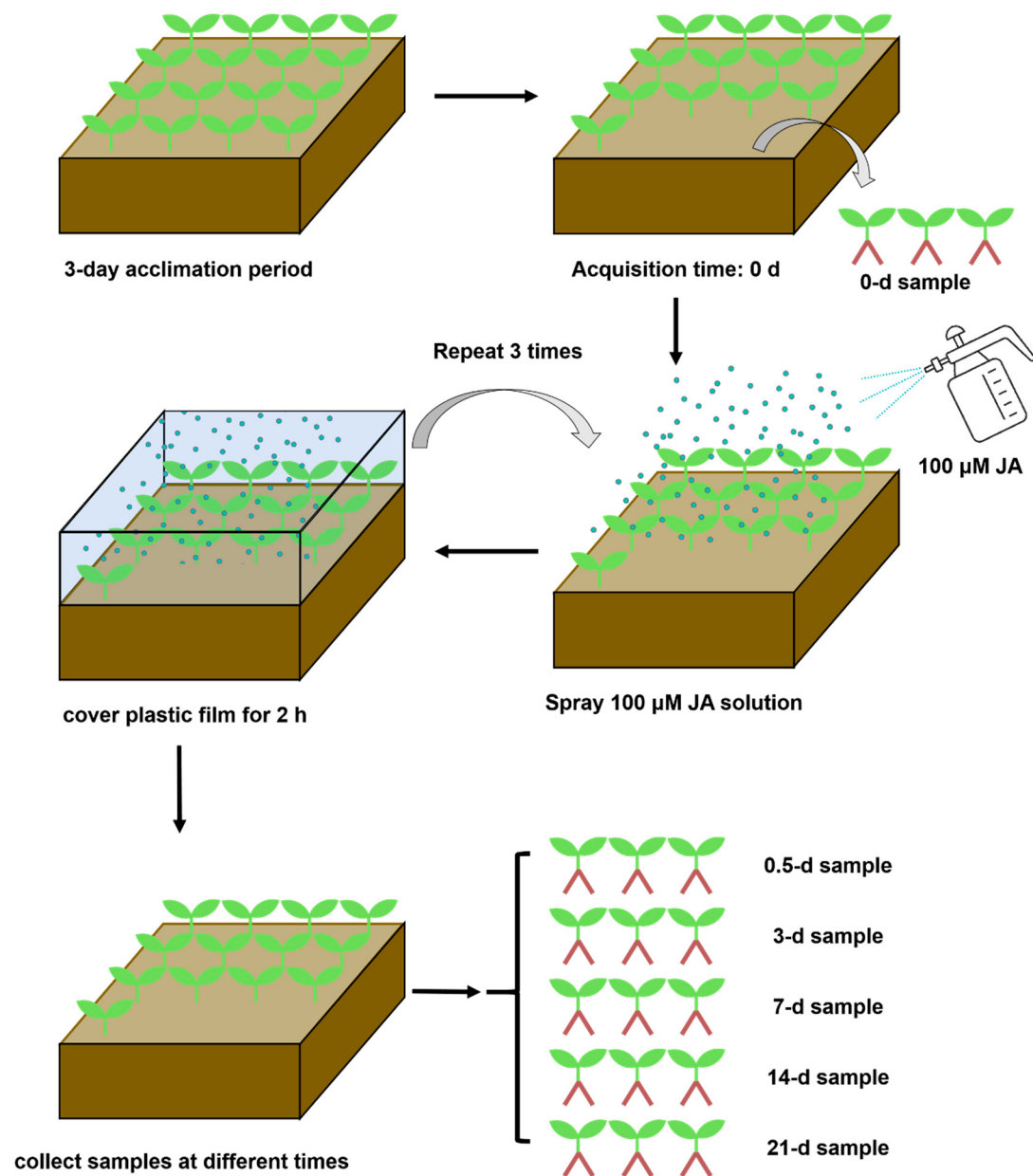

**Figure S1.** JA treatment of 3-year-old *S. hexandrum* plants and sampling methods

Supplement: Supplementary file 1 [file plants-14-01001-s001.zip › Supplementary figure.pdf]
